# Complete Genome Sequences of Dengue Virus Type 2 Strains from Kilifi, Kenya

**DOI:** 10.1128/MRA.01566-18

**Published:** 2019-01-24

**Authors:** Everlyn Kamau, Charles N. Agoti, Joyce M. Ngoi, Zaydah R. de Laurent, John Gitonga, Matthew Cotten, My V. T. Phan, D. James Nokes, Eric Delwart, Eduard Sanders, George M. Warimwe

**Affiliations:** a KEMRI-Wellcome Trust Research Programme, Kilifi, Kenya; b Department of Viroscience, Erasmus MC, Rotterdam, The Netherlands; c School of Life Sciences, Zeeman Institute, University of Warwick, Coventry, United Kingdom; d Blood Systems Research Institute, University of California, San Francisco, California, USA; e Centre for Tropical Medicine and Global Health, University of Oxford, Oxford, United Kingdom; University of Maryland School of Medicine

## Abstract

Dengue infection remains poorly characterized in Africa and little is known regarding its associated viral genetic diversity. Here, we report dengue virus type 2 (DENV-2) sequence data from 10 clinical samples, including 5 complete genome sequences of the cosmopolitan genotype, obtained from febrile adults seeking outpatient care in coastal Kenya.

## ANNOUNCEMENT

Dengue virus (DENV) is a mosquito-borne, enveloped flavivirus with an RNA genome of ∼10.7 kb (1). DENV, further classified into four distinct serotypes (DENV-1 to DENV-4), is widespread globally, causing an estimated 390 million infections yearly (2, 3). A licensed vaccine, Dengvaxia, is available for use in individuals with proof of prior infections (4), while other candidate vaccines are in development (5). Although an estimated >60 million DENV infections occur in Africa annually (1), DENV epidemiology is poorly characterized, including the circulating viral genetic diversity (6–8). Studies reporting new DENV genomes have the potential to facilitate the development of DENV vaccines. Here, we report 5 complete and 5 partial DENV-2 genome sequences identified from plasma samples of patients participating in a cross-sectional study on the burden of DENV and chikungunya virus in coastal Kenya (9). A detailed description of the study design is provided elsewhere (9, 10). The participants, aged 18 to 35 years, were seeking care at health facilities in Mtwapa, Kilifi County between February 2014 and January 2015. DENV detection used the CDC DENV-1 to -4 real-time reverse transcriptase PCR (RT-PCR) assay kit (catalog number KK0128). All participants provided written informed consent, and the study protocol was approved by the Scientific and Ethics Review Unit (Kenya) and the University of Oxford Tropical Research Ethics Committee in United Kingdom.

Sequencing was performed as previously described (11, 12). Total nucleic acid was extracted from 10 DENV-positive samples using the TRIzol LS reagent (Invitrogen) and DNase treated (TURBO DNase; Invitrogen). cDNA was synthesized using SuperScript III reverse transcriptase (Invitrogen) and nonribosomal hexanucleotide primers with reduced rRNA targets. Second-strand synthesis was performed using a Klenow fragment (New England BioLabs). Standard Illumina libraries were prepared using the Nextera XT kit (Illumina), and paired-end sequencing (2 × 250 bp) was performed with the MiSeq reagent v2 kit (Illumina). Short reads were filtered for quality using quality assessment of short read (QUASR) v.7.03 (13) and *de novo* assembled using SPAdes v.3.11 (14), and *N*_50_ values were determined using QUAST v.3.2.0 (15). Mean genome coverage was calculated by mapping short reads onto individual assemblies with Bowtie2 v.2.3.4.3 (16), followed by using the SAMtools v.1.9 sort and index functions (17) on the aligned bam files, and then using the bedtools v.2.27.0 genomecov function (18) for generating the coverage statistics.

Sequencing and data assembly results and parameters are shown in Table 1. Full-length DENV genomes (>10 kb) were obtained from 5 samples while the remaining 5 samples had genomes between 2,407 and 8,372 nucleotides. Maximum likelihood phylogeny of envelope gene sequences, together with representative sequences of known DENV genotypes, classified the strains as the DENV-2 cosmopolitan genotype (19). Sequence annotation of the full-length genomes using Geneious R8.1.5 identified the expected polyprotein (10,176 nucleotides [nt], 3,392 amino acids) that yields 3 structural proteins, 7 nonstructural proteins, and a flanking 214-nt 3′ untranslated region (UTR) segment. No deletions, insertions, or premature stop codons were identified within the polyprotein-coding region. All five genomes showed a high level (>99.4%) of nucleotide similarity. Our results demonstrate an application for unbiased next-generation sequencing (NGS) without pathogen-specific enrichment. The new data provide a useful reference for the design of local diagnostics and for studies aimed at understanding DENV evolution and transmission in Kenya.

**TABLE 1:** Sequencing results and data assembly metrics for the partial and complete DENV-2 genomes from Kilifi, Kenya^*a*^

Strain	Total no. of reads^*b*^	No. of contigs^*c*^	*N*_50_ value	Genome length (bp)	G+C content (%)	Mean coverage (×)	GenBank accession no.	BioSample accession no.
DENV2/KLF/001/2014	196,326	637	1,161	10,093	45.9	1,023.3	MH456892	SAMN10606308
DENV2/KLF/004/2014	22,182	59	10,632	10,173	46.1	236.45	MH456893	SAMN10606311
DENV2/KLF/006/2014	2,505,710	5,989	613	8,372	45.7	3,452.32	MH456894	SAMN10606313
DENV2/KLF/005/2014	231,334	282	1,315	10,173	46.0	2,981.43	MH456895	SAMN10606312
DENV2/KLF/009/2014	4,963,410	22,485	791	4,272	45.5	3,324.8	MH456896	SAMN10606316
DENV2/KLF/002/2014	4,399,878	15,101	620	10,173	46.0	6,172.7	MH456897	SAMN10606309
DENV2/KLF/003/2014	195,090	208	825	10,173	46.1	3,589.76	MH456898	SAMN10606310
DENV2/KLF/008/2014	210,582	106	2,464	7,859	46.2	2,290.83	MH456899	SAMN10606315
DENV2/KLF/007/2014	75,768	359	791	2,407	45.2	451.82	MH456900	SAMN10606314

a QUASR parameters, “-d –q –l 125 –m 30”; SPAdes parameters, “–careful”; QUAST parameters, “minimum contig length: 500, ambiguity: one, threshold for extensive mis-assembly size: 1000”; Bowtie2: parameters, “-q –S –local.”

b Short read length ranged from 35 to 250 bases.

c Contig refers to contiguous length of genomic sequence in which the order of bases is known to a high confidence level.

### Data availability.

The assembled sequences for full (five) and partial (four) genomes are available in the GenBank nucleotide database under accession numbers MH456892 to MH456900. The raw data are available in the NCBI SRA archive under BioProject accession number PRJNA510506.
